# Supplementary figures and images for: Trypanosome KKIP1 Dynamically Links the Inner Kinetochore to a Kinetoplastid Outer Kinetochore Complex (part 2 of 2)
Source: Front Cell Infect Microbiol. 2021 Mar 23;11:641174. doi: 10.3389/fcimb.2021.641174 (PMC8023272; doi:10.3389/fcimb.2021.641174)

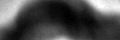

Supplement: Supplementary file 1 [file DataSheet_1.zip › SupplementaryFile3/RotatedCells/190115_K1.K1-Y.A.137.1.tif]

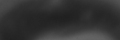

Supplement: Supplementary file 1 [file DataSheet_1.zip › SupplementaryFile3/RotatedCells/190104_K1.Y-K1.M.069.1.tif]

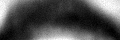

Supplement: Supplementary file 1 [file DataSheet_1.zip › SupplementaryFile3/RotatedCells/190115_K1.Y-K1.M.094.1.tif]

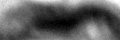

Supplement: Supplementary file 1 [file DataSheet_1.zip › SupplementaryFile3/RotatedCells/190115_K1.Y-K1.M.030.1.tif]

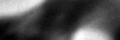

Supplement: Supplementary file 1 [file DataSheet_1.zip › SupplementaryFile3/RotatedCells/190115_K1.Y-K1.M.071.1.tif]

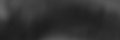

Supplement: Supplementary file 1 [file DataSheet_1.zip › SupplementaryFile3/RotatedCells/190107_K1.Y-K1.M.109.1.tif]

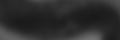

Supplement: Supplementary file 1 [file DataSheet_1.zip › SupplementaryFile3/RotatedCells/190107_K1.Y-K1.A.146.1.tif]

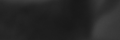

Supplement: Supplementary file 1 [file DataSheet_1.zip › SupplementaryFile3/RotatedCells/181126_K1.Y-K1.M.036.1.tif]

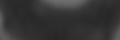

Supplement: Supplementary file 1 [file DataSheet_1.zip › SupplementaryFile3/RotatedCells/190104_K1.Y-K1.A.022.1.tif]

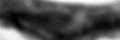

Supplement: Supplementary file 1 [file DataSheet_1.zip › SupplementaryFile3/RotatedCells/181126_K1.K1-Y.M.030.1.tif]

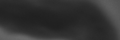

Supplement: Supplementary file 1 [file DataSheet_1.zip › SupplementaryFile3/RotatedCells/190104_K1.Y-K1.M.009.1.tif]

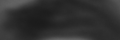

Supplement: Supplementary file 1 [file DataSheet_1.zip › SupplementaryFile3/RotatedCells/190107_K1.K1-Y.A.058.1.tif]

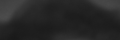

Supplement: Supplementary file 1 [file DataSheet_1.zip › SupplementaryFile3/RotatedCells/181126_K1.K1-Y.M.010.1.tif]

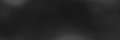

Supplement: Supplementary file 1 [file DataSheet_1.zip › SupplementaryFile3/RotatedCells/181126_K1.K1-Y.M.009.1.tif]

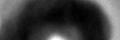

Supplement: Supplementary file 1 [file DataSheet_1.zip › SupplementaryFile3/RotatedCells/190115_K1.K1-Y.M.146.1.tif]

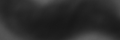

Supplement: Supplementary file 1 [file DataSheet_1.zip › SupplementaryFile3/RotatedCells/190107_K1.K1-Y.M.073.1.tif]

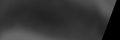

Supplement: Supplementary file 1 [file DataSheet_1.zip › SupplementaryFile3/RotatedCells/190104_K1.Y-K1.M.112.1.tif]

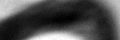

Supplement: Supplementary file 1 [file DataSheet_1.zip › SupplementaryFile3/RotatedCells/190115_K1.Y-K1.A.038.1.tif]

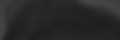

Supplement: Supplementary file 1 [file DataSheet_1.zip › SupplementaryFile3/RotatedCells/181126_K1.K1-Y.M.026.1.tif]

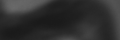

Supplement: Supplementary file 1 [file DataSheet_1.zip › SupplementaryFile3/RotatedCells/190107_K1.Y-K1.M.134.1.tif]

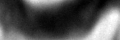

Supplement: Supplementary file 1 [file DataSheet_1.zip › SupplementaryFile3/RotatedCells/190115_K1.K1-Y.M.105.1.tif]

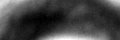

Supplement: Supplementary file 1 [file DataSheet_1.zip › SupplementaryFile3/RotatedCells/190115_K1.Y-K1.A.078.1.tif]

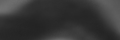

Supplement: Supplementary file 1 [file DataSheet_1.zip › SupplementaryFile3/RotatedCells/190107_K1.Y-K1.M.052.1.tif]

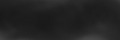

Supplement: Supplementary file 1 [file DataSheet_1.zip › SupplementaryFile3/RotatedCells/181126_K1.K1-Y.M.007.1.tif]

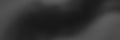

Supplement: Supplementary file 1 [file DataSheet_1.zip › SupplementaryFile3/RotatedCells/190107_K1.K1-Y.M.019.1.tif]

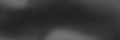

Supplement: Supplementary file 1 [file DataSheet_1.zip › SupplementaryFile3/RotatedCells/190104_K1.K1-Y.A.042.1.tif]

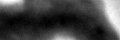

Supplement: Supplementary file 1 [file DataSheet_1.zip › SupplementaryFile3/RotatedCells/181126_K1.Y-K1.M.023.1.tif]

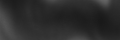

Supplement: Supplementary file 1 [file DataSheet_1.zip › SupplementaryFile3/RotatedCells/190107_K1.Y-K1.A.010.1.tif]

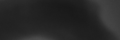

Supplement: Supplementary file 1 [file DataSheet_1.zip › SupplementaryFile3/RotatedCells/181126_K1.K1-Y.M.013.1.tif]

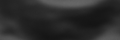

Supplement: Supplementary file 1 [file DataSheet_1.zip › SupplementaryFile3/RotatedCells/190107_K1.K1-Y.A.065.1.tif]

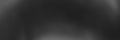

Supplement: Supplementary file 1 [file DataSheet_1.zip › SupplementaryFile3/RotatedCells/190104_K1.Y-K1.M.108.1.tif]

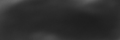

Supplement: Supplementary file 1 [file DataSheet_1.zip › SupplementaryFile3/RotatedCells/190104_K1.Y-K1.A.005.1.tif]
