# Supplementary figures and images for: Trypanosome KKIP1 Dynamically Links the Inner Kinetochore to a Kinetoplastid Outer Kinetochore Complex (part 1 of 2)
Source: Front Cell Infect Microbiol. 2021 Mar 23;11:641174. doi: 10.3389/fcimb.2021.641174 (PMC8023272; doi:10.3389/fcimb.2021.641174)

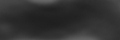

Supplement: Supplementary file 1 [file DataSheet_1.zip › SupplementaryFile3/RotatedCells/190104_K1.K1-Y.M.034.1.tif]

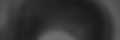

Supplement: Supplementary file 1 [file DataSheet_1.zip › SupplementaryFile3/RotatedCells/190107_K1.K1-Y.A.044.1.tif]

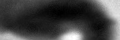

Supplement: Supplementary file 1 [file DataSheet_1.zip › SupplementaryFile3/RotatedCells/190115_K1.K1-Y.A.119.1.tif]

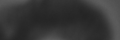

Supplement: Supplementary file 1 [file DataSheet_1.zip › SupplementaryFile3/RotatedCells/190104_K1.Y-K1.M.040.1.tif]

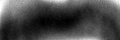

Supplement: Supplementary file 1 [file DataSheet_1.zip › SupplementaryFile3/RotatedCells/190115_K1.K1-Y.M.055.1.tif]

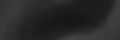

Supplement: Supplementary file 1 [file DataSheet_1.zip › SupplementaryFile3/RotatedCells/181126_K1.Y-K1.M.018.1.tif]

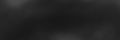

Supplement: Supplementary file 1 [file DataSheet_1.zip › SupplementaryFile3/RotatedCells/181126_K1.K1-Y.A.003.1.tif]

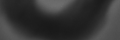

Supplement: Supplementary file 1 [file DataSheet_1.zip › SupplementaryFile3/RotatedCells/190107_K1.K1-Y.M.109.1.tif]

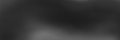

Supplement: Supplementary file 1 [file DataSheet_1.zip › SupplementaryFile3/RotatedCells/190104_K1.Y-K1.M.039.1.tif]

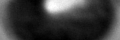

Supplement: Supplementary file 1 [file DataSheet_1.zip › SupplementaryFile3/RotatedCells/190115_K1.K1-Y.A.112.1.tif]

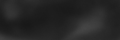

Supplement: Supplementary file 1 [file DataSheet_1.zip › SupplementaryFile3/RotatedCells/181126_K1.K1-Y.M.005.1.tif]

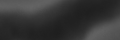

Supplement: Supplementary file 1 [file DataSheet_1.zip › SupplementaryFile3/RotatedCells/190104_K1.Y-K1.M.085.1.tif]

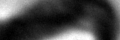

Supplement: Supplementary file 1 [file DataSheet_1.zip › SupplementaryFile3/RotatedCells/190115_K1.Y-K1.M.013.1.tif]

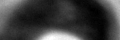

Supplement: Supplementary file 1 [file DataSheet_1.zip › SupplementaryFile3/RotatedCells/190115_K1.K1-Y.A.036.1.tif]

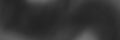

Supplement: Supplementary file 1 [file DataSheet_1.zip › SupplementaryFile3/RotatedCells/190107_K1.Y-K1.A.141.1.tif]

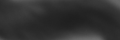

Supplement: Supplementary file 1 [file DataSheet_1.zip › SupplementaryFile3/RotatedCells/190104_K1.Y-K1.M.016.1.tif]

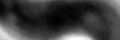

Supplement: Supplementary file 1 [file DataSheet_1.zip › SupplementaryFile3/RotatedCells/190115_K1.K1-Y.M.030.1.tif]

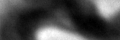

Supplement: Supplementary file 1 [file DataSheet_1.zip › SupplementaryFile3/RotatedCells/190115_K1.Y-K1.A.112.1.tif]

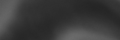

Supplement: Supplementary file 1 [file DataSheet_1.zip › SupplementaryFile3/RotatedCells/190104_K1.Y-K1.M.057.1.tif]

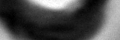

Supplement: Supplementary file 1 [file DataSheet_1.zip › SupplementaryFile3/RotatedCells/190115_K1.K1-Y.M.126.1.tif]

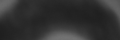

Supplement: Supplementary file 1 [file DataSheet_1.zip › SupplementaryFile3/RotatedCells/190107_K1.Y-K1.A.041.1.tif]

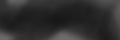

Supplement: Supplementary file 1 [file DataSheet_1.zip › SupplementaryFile3/RotatedCells/190107_K1.K1-Y.A.105.1.tif]

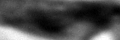

Supplement: Supplementary file 1 [file DataSheet_1.zip › SupplementaryFile3/RotatedCells/190115_K1.K1-Y.A.130.1.tif]

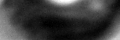

Supplement: Supplementary file 1 [file DataSheet_1.zip › SupplementaryFile3/RotatedCells/190115_K1.Y-K1.A.077.1.tif]

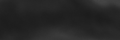

Supplement: Supplementary file 1 [file DataSheet_1.zip › SupplementaryFile3/RotatedCells/181126_K1.Y-K1.M.007.1.tif]

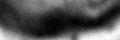

Supplement: Supplementary file 1 [file DataSheet_1.zip › SupplementaryFile3/RotatedCells/190115_K1.Y-K1.M.254.1.tif]

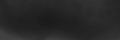

Supplement: Supplementary file 1 [file DataSheet_1.zip › SupplementaryFile3/RotatedCells/181126_K1.Y-K1.M.004.1.tif]

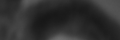

Supplement: Supplementary file 1 [file DataSheet_1.zip › SupplementaryFile3/RotatedCells/190107_K1.K1-Y.M.068.1.tif]

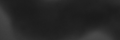

Supplement: Supplementary file 1 [file DataSheet_1.zip › SupplementaryFile3/RotatedCells/181126_K1.K1-Y.A.034.1.tif]

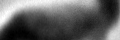

Supplement: Supplementary file 1 [file DataSheet_1.zip › SupplementaryFile3/RotatedCells/190115_K1.K1-Y.M.049.1.tif]

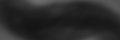

Supplement: Supplementary file 1 [file DataSheet_1.zip › SupplementaryFile3/RotatedCells/190107_K1.K1-Y.A.080.1.tif]

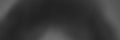

Supplement: Supplementary file 1 [file DataSheet_1.zip › SupplementaryFile3/RotatedCells/190104_K1.Y-K1.M.043.1.tif]

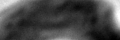

Supplement: Supplementary file 1 [file DataSheet_1.zip › SupplementaryFile3/RotatedCells/181126_K1.Y-K1.A.045.1.tif]

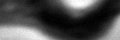

Supplement: Supplementary file 1 [file DataSheet_1.zip › SupplementaryFile3/RotatedCells/190115_K1.Y-K1.M.085.1.tif]

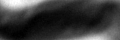

Supplement: Supplementary file 1 [file DataSheet_1.zip › SupplementaryFile3/RotatedCells/190115_K1.Y-K1.A.014.1.tif]

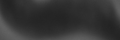

Supplement: Supplementary file 1 [file DataSheet_1.zip › SupplementaryFile3/RotatedCells/190104_K1.Y-K1.M.061.1.tif]

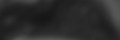

Supplement: Supplementary file 1 [file DataSheet_1.zip › SupplementaryFile3/RotatedCells/190107_K1.K1-Y.M.027.1.tif]

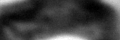

Supplement: Supplementary file 1 [file DataSheet_1.zip › SupplementaryFile3/RotatedCells/190115_K1.Y-K1.A.004.1.tif]

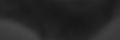

Supplement: Supplementary file 1 [file DataSheet_1.zip › SupplementaryFile3/RotatedCells/181126_K1.Y-K1.M.034.1.tif]

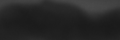

Supplement: Supplementary file 1 [file DataSheet_1.zip › SupplementaryFile3/RotatedCells/181126_K1.K1-Y.A.015.1.tif]

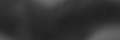

Supplement: Supplementary file 1 [file DataSheet_1.zip › SupplementaryFile3/RotatedCells/190104_K1.Y-K1.A.045.1.tif]

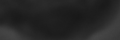

Supplement: Supplementary file 1 [file DataSheet_1.zip › SupplementaryFile3/RotatedCells/181126_K1.K1-Y.M.029.1.tif]

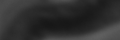

Supplement: Supplementary file 1 [file DataSheet_1.zip › SupplementaryFile3/RotatedCells/190107_K1.K1-Y.A.009.1.tif]

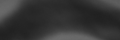

Supplement: Supplementary file 1 [file DataSheet_1.zip › SupplementaryFile3/RotatedCells/190104_K1.K1-Y.A.027.1.tif]

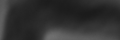

Supplement: Supplementary file 1 [file DataSheet_1.zip › SupplementaryFile3/RotatedCells/190107_K1.Y-K1.A.118.1.tif]

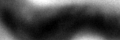

Supplement: Supplementary file 1 [file DataSheet_1.zip › SupplementaryFile3/RotatedCells/190115_K1.K1-Y.M.142.1.tif]

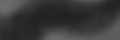

Supplement: Supplementary file 1 [file DataSheet_1.zip › SupplementaryFile3/RotatedCells/190107_K1.Y-K1.M.026.1.tif]

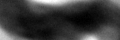

Supplement: Supplementary file 1 [file DataSheet_1.zip › SupplementaryFile3/RotatedCells/190115_K1.K1-Y.A.143.1.tif]

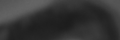

Supplement: Supplementary file 1 [file DataSheet_1.zip › SupplementaryFile3/RotatedCells/190107_K1.K1-Y.M.081.1.tif]

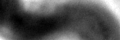

Supplement: Supplementary file 1 [file DataSheet_1.zip › SupplementaryFile3/RotatedCells/181126_K1.K1-Y.M.006.1.tif]

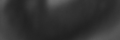

Supplement: Supplementary file 1 [file DataSheet_1.zip › SupplementaryFile3/RotatedCells/190107_K1.Y-K1.M.112.1.tif]

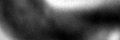

Supplement: Supplementary file 1 [file DataSheet_1.zip › SupplementaryFile3/RotatedCells/190115_K1.K1-Y.A.059.1.tif]

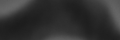

Supplement: Supplementary file 1 [file DataSheet_1.zip › SupplementaryFile3/RotatedCells/190104_K1.Y-K1.A.107.1.tif]

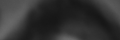

Supplement: Supplementary file 1 [file DataSheet_1.zip › SupplementaryFile3/RotatedCells/190107_K1.K1-Y.M.090.1.tif]

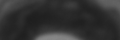

Supplement: Supplementary file 1 [file DataSheet_1.zip › SupplementaryFile3/RotatedCells/190107_K1.Y-K1.A.058.1.tif]

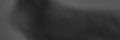

Supplement: Supplementary file 1 [file DataSheet_1.zip › SupplementaryFile3/RotatedCells/190107_K1.Y-K1.M.119.1.tif]

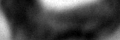

Supplement: Supplementary file 1 [file DataSheet_1.zip › SupplementaryFile3/RotatedCells/190115_K1.Y-K1.A.093.1.tif]

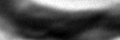

Supplement: Supplementary file 1 [file DataSheet_1.zip › SupplementaryFile3/RotatedCells/190115_K1.Y-K1.A.001.1.tif]

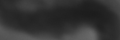

Supplement: Supplementary file 1 [file DataSheet_1.zip › SupplementaryFile3/RotatedCells/190104_K1.K1-Y.A.016.1.tif]

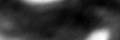

Supplement: Supplementary file 1 [file DataSheet_1.zip › SupplementaryFile3/RotatedCells/190115_K1.K1-Y.A.014.1.tif]

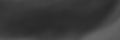

Supplement: Supplementary file 1 [file DataSheet_1.zip › SupplementaryFile3/RotatedCells/190107_K1.Y-K1.M.028.1.tif]

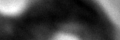

Supplement: Supplementary file 1 [file DataSheet_1.zip › SupplementaryFile3/RotatedCells/190115_K1.K1-Y.A.057.1.tif]

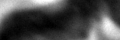

Supplement: Supplementary file 1 [file DataSheet_1.zip › SupplementaryFile3/RotatedCells/190115_K1.Y-K1.M.008.1.tif]

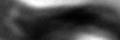

Supplement: Supplementary file 1 [file DataSheet_1.zip › SupplementaryFile3/RotatedCells/190115_K1.Y-K1.M.158.1.tif]

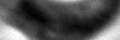

Supplement: Supplementary file 1 [file DataSheet_1.zip › SupplementaryFile3/RotatedCells/190115_K1.Y-K1.M.052.1.tif]

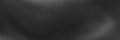

Supplement: Supplementary file 1 [file DataSheet_1.zip › SupplementaryFile3/RotatedCells/190104_K1.Y-K1.A.126.1.tif]

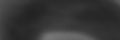

Supplement: Supplementary file 1 [file DataSheet_1.zip › SupplementaryFile3/RotatedCells/190107_K1.Y-K1.A.050.1.tif]

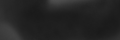

Supplement: Supplementary file 1 [file DataSheet_1.zip › SupplementaryFile3/RotatedCells/181126_K1.Y-K1.A.011.1.tif]

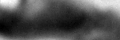

Supplement: Supplementary file 1 [file DataSheet_1.zip › SupplementaryFile3/RotatedCells/190115_K1.Y-K1.A.089.1.tif]

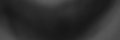

Supplement: Supplementary file 1 [file DataSheet_1.zip › SupplementaryFile3/RotatedCells/190107_K1.K1-Y.M.086.1.tif]

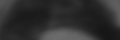

Supplement: Supplementary file 1 [file DataSheet_1.zip › SupplementaryFile3/RotatedCells/190107_K1.K1-Y.A.045.1.tif]

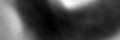

Supplement: Supplementary file 1 [file DataSheet_1.zip › SupplementaryFile3/RotatedCells/190115_K1.K1-Y.M.117.1.tif]

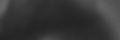

Supplement: Supplementary file 1 [file DataSheet_1.zip › SupplementaryFile3/RotatedCells/190104_K1.Y-K1.A.028.1.tif]

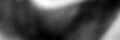

Supplement: Supplementary file 1 [file DataSheet_1.zip › SupplementaryFile3/RotatedCells/190115_K1.K1-Y.A.083.1.tif]

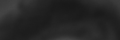

Supplement: Supplementary file 1 [file DataSheet_1.zip › SupplementaryFile3/RotatedCells/181126_K1.K1-Y.A.022.1.tif]

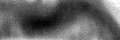

Supplement: Supplementary file 1 [file DataSheet_1.zip › SupplementaryFile3/RotatedCells/190115_K1.Y-K1.M.058.1.tif]

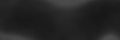

Supplement: Supplementary file 1 [file DataSheet_1.zip › SupplementaryFile3/RotatedCells/181126_K1.K1-Y.A.001.1.tif]

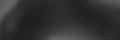

Supplement: Supplementary file 1 [file DataSheet_1.zip › SupplementaryFile3/RotatedCells/190104_K1.Y-K1.A.111.1.tif]

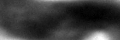

Supplement: Supplementary file 1 [file DataSheet_1.zip › SupplementaryFile3/RotatedCells/190115_K1.K1-Y.M.016.1.tif]

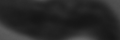

Supplement: Supplementary file 1 [file DataSheet_1.zip › SupplementaryFile3/RotatedCells/190107_K1.K1-Y.A.095.1.tif]

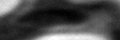

Supplement: Supplementary file 1 [file DataSheet_1.zip › SupplementaryFile3/RotatedCells/190115_K1.Y-K1.A.082.1.tif]

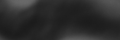

Supplement: Supplementary file 1 [file DataSheet_1.zip › SupplementaryFile3/RotatedCells/190107_K1.Y-K1.A.126.1.tif]

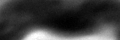

Supplement: Supplementary file 1 [file DataSheet_1.zip › SupplementaryFile3/RotatedCells/190115_K1.K1-Y.M.139.1.tif]

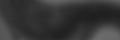

Supplement: Supplementary file 1 [file DataSheet_1.zip › SupplementaryFile3/RotatedCells/190107_K1.Y-K1.A.049.1.tif]

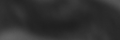

Supplement: Supplementary file 1 [file DataSheet_1.zip › SupplementaryFile3/RotatedCells/190107_K1.Y-K1.M.022.1.tif]

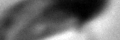

Supplement: Supplementary file 1 [file DataSheet_1.zip › SupplementaryFile3/RotatedCells/190115_K1.K1-Y.M.107.1.tif]

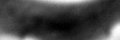

Supplement: Supplementary file 1 [file DataSheet_1.zip › SupplementaryFile3/RotatedCells/181126_K1.Y-K1.M.019.1.tif]

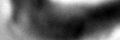

Supplement: Supplementary file 1 [file DataSheet_1.zip › SupplementaryFile3/RotatedCells/190115_K1.Y-K1.M.002.1.tif]

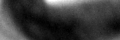

Supplement: Supplementary file 1 [file DataSheet_1.zip › SupplementaryFile3/RotatedCells/190115_K1.Y-K1.M.062.1.tif]

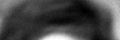

Supplement: Supplementary file 1 [file DataSheet_1.zip › SupplementaryFile3/RotatedCells/190115_K1.Y-K1.A.156.1.tif]

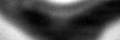

Supplement: Supplementary file 1 [file DataSheet_1.zip › SupplementaryFile3/RotatedCells/190115_K1.Y-K1.A.080.1.tif]

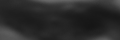

Supplement: Supplementary file 1 [file DataSheet_1.zip › SupplementaryFile3/RotatedCells/190107_K1.K1-Y.A.053.1.tif]

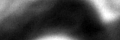

Supplement: Supplementary file 1 [file DataSheet_1.zip › SupplementaryFile3/RotatedCells/190115_K1.K1-Y.M.134.1.tif]

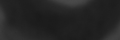

Supplement: Supplementary file 1 [file DataSheet_1.zip › SupplementaryFile3/RotatedCells/181126_K1.Y-K1.A.022.1.tif]

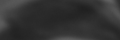

Supplement: Supplementary file 1 [file DataSheet_1.zip › SupplementaryFile3/RotatedCells/190107_K1.Y-K1.M.044.1.tif]

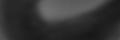

Supplement: Supplementary file 1 [file DataSheet_1.zip › SupplementaryFile3/RotatedCells/190107_K1.K1-Y.A.093.1.tif]

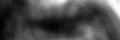

Supplement: Supplementary file 1 [file DataSheet_1.zip › SupplementaryFile3/RotatedCells/181126_K1.Y-K1.M.045.1.tif]

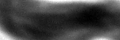

Supplement: Supplementary file 1 [file DataSheet_1.zip › SupplementaryFile3/RotatedCells/190115_K1.K1-Y.A.020.1.tif]

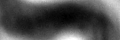

Supplement: Supplementary file 1 [file DataSheet_1.zip › SupplementaryFile3/RotatedCells/190115_K1.Y-K1.M.045.1.tif]

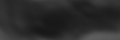

Supplement: Supplementary file 1 [file DataSheet_1.zip › SupplementaryFile3/RotatedCells/190107_K1.K1-Y.M.123.1.tif]
