# Supplementary material for: Trypanosome KKIP1 Dynamically Links the Inner Kinetochore to a Kinetoplastid Outer Kinetochore Complex
Source: Front Cell Infect Microbiol. 2021 Mar 23;11:641174. doi: 10.3389/fcimb.2021.641174 (PMC8023272; doi:10.3389/fcimb.2021.641174)
Supplement: Supplementary file 2 [file DataSheet_2.pdf]

Supplementary Material

Supplementary Data

Supplementary File 1. Normalized spectral index quantitation for 1582 distinguishable trypanosome proteins detected with ≥2 peptides across all immuno-purification experiments. Original data are available at the PRIDE partner repository with the dataset identifier PXD015100.

Supplementary File 2. Primers used for gene targeting in this study.

Supplementary File 3. Images, centroid positions and source code used for analysis of label separation in Figure 5. Images have been rotated to place spindle along x-axis and cropped to regions of 7.74x2.58 µm around the spindle center, but are otherwise unprocessed. Channels: phase contrast, mSt-KKT2, YFP-KKIP1/KKIP1-YFP, mTu-KKIP3, DAPI.

Supplementary Figures

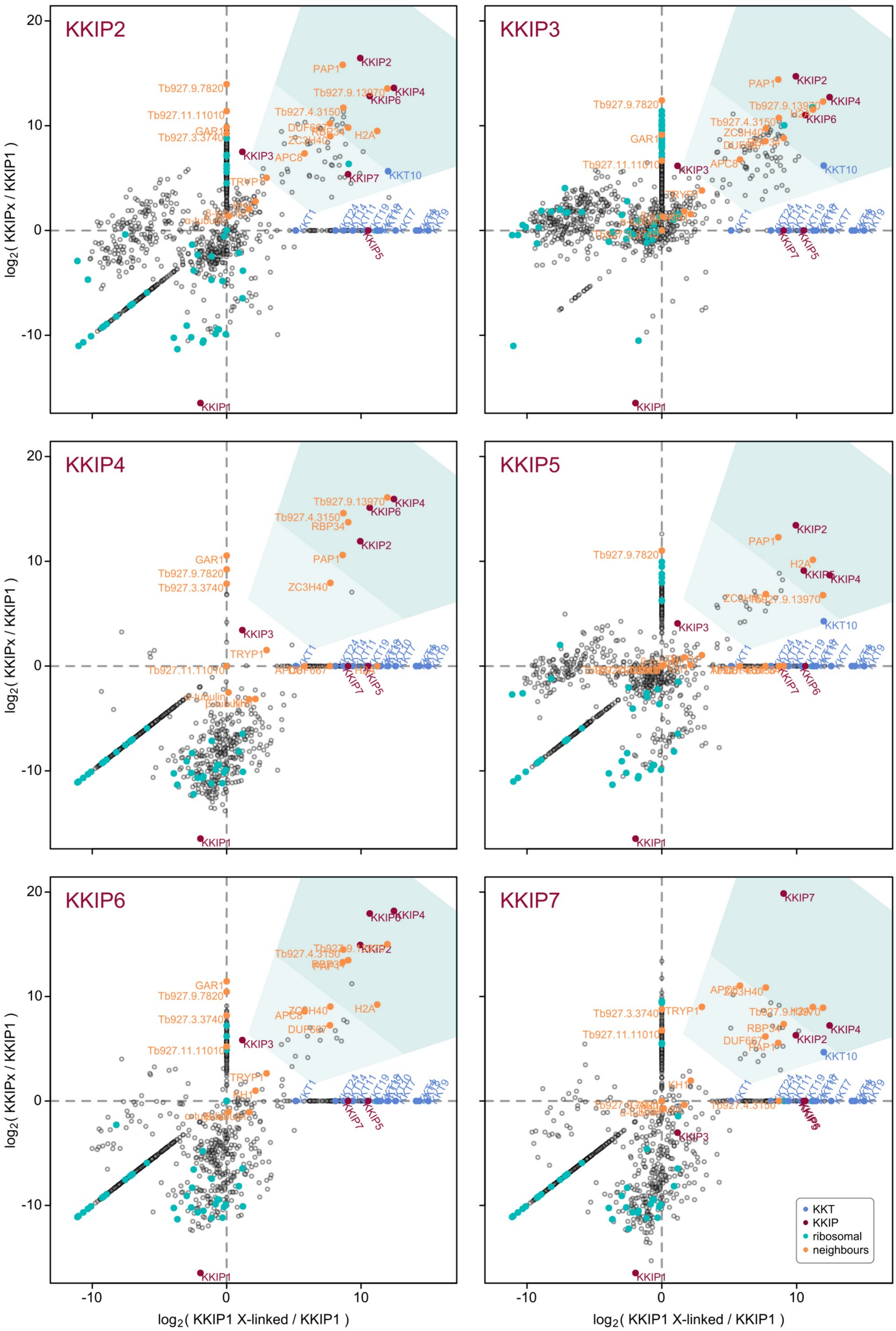
KKIP2
KKIP3
KKIP4
KKIP5
KKIP6
KKIP7
log2(KKIPx / KKIP1)
log2(KKIP1 X-linked / KKIP1)
20
10
0
-10
10
KKT
KKIP
ribosomal
neighbours
PAP1
KKIP2
Tb927.9.7820
Tb927.9.13970
KKIP4
KKIP6
Tb927.11.11010
Tb927.4.3150
GAR1
Tb927.3.3740
ZC3H40
RBP34
DUF667
H2A
KKIP3
APC8
TRYP1
KKIP7
KKT10
KKT1
KKIP5
KKIP1

Supplementary Figure S1. Multiple proteins are identified across KKIP immuno-purifications. Label-free semi-quantitative mass spectrometry showing relative enrichment of proteins co-purified with KKIP1-KKIP7 against amounts detected co-purifying with KKIP1 under standard (non cross-linking) or cross-linking conditions (D’Archivio and Wickstead, 2017). Proteins identified as ribosomal, KKTs and KKIPs are highlighted, in addition to proteins highly enriched in multiple experiments (neighbors). For display, intensity of proteins not detected in a specific immunoprecipitation are set to an arbitrary minimum value.

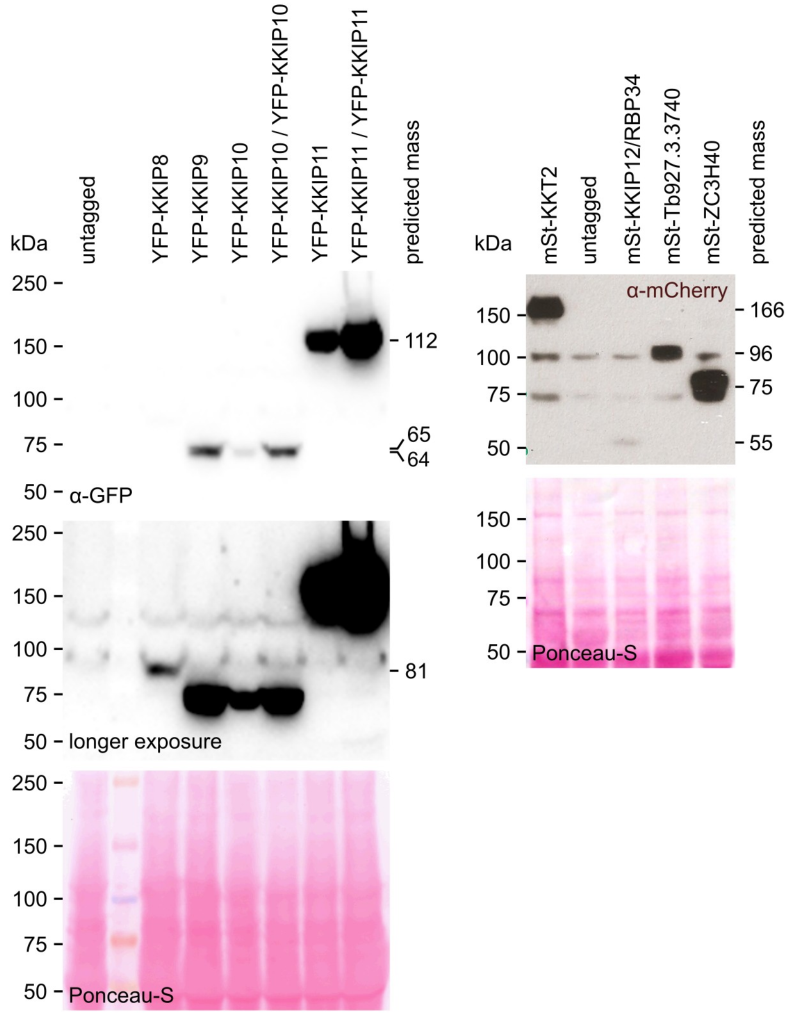

Supplementary Figure S2. Confirmation of tagging and expression of KKIP or KKIP-interacting proteins. Immunoblots of whole cell lysates from cells expressing YFP- or mStrawberry- (mSt-) tagged KKIP or KKIP-interacting proteins from their endogenous loci. Ponceau-S staining of total protein on the membrane is shown as a control for loading.

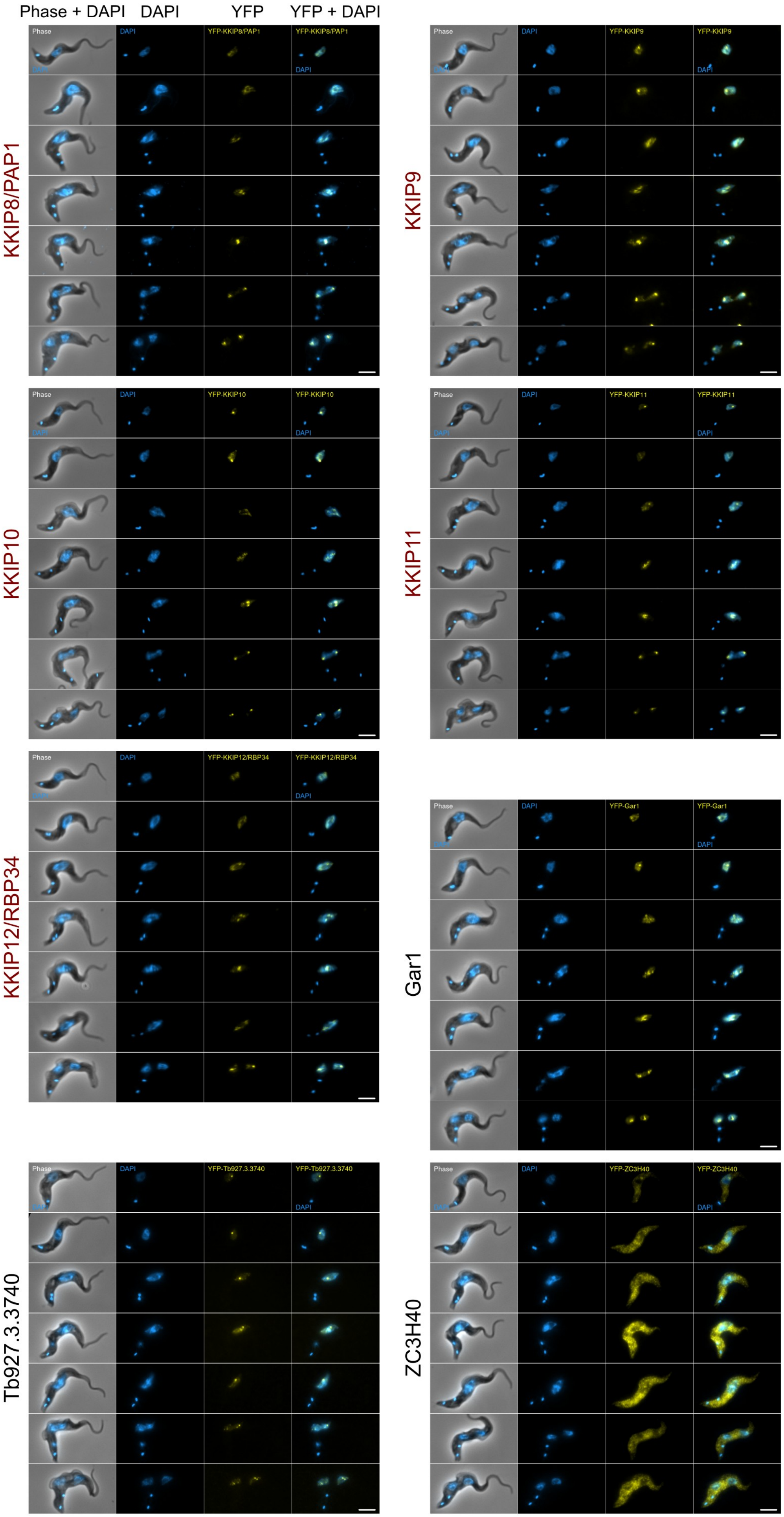

Phase + DAPI
DAPI
YFP
YFP + DAPI
KKIP8/PAP1
Phase
DAPI
YFP-KKIP8/PAP1
KKIP9
YFP-KKIP9
KKIP10
YFP-KKIP10
KKIP11
YFP-KKIP11
KKIP12/RBP34
YFP-KKIP12/RBP34
Gar1
YFP-Gar1
Tb927.3.3740
YFP-Tb927.3.3740
ZC3H40
YFP-ZC3H40

Supplementary Figure S3. Spatio-temporal localization of newly identified kinetoplastid kinetochore components through the trypanosome cell cycle. Representative images of cells arranged by inferred position in the cell cycle (G1 to cytokinesis from top to bottom) are shown. Native fluorescence from YFP (yellow), in addition to counter-staining of DNA with DAPI (cyan) and phase contrast images. Scale bars: 2 μm.

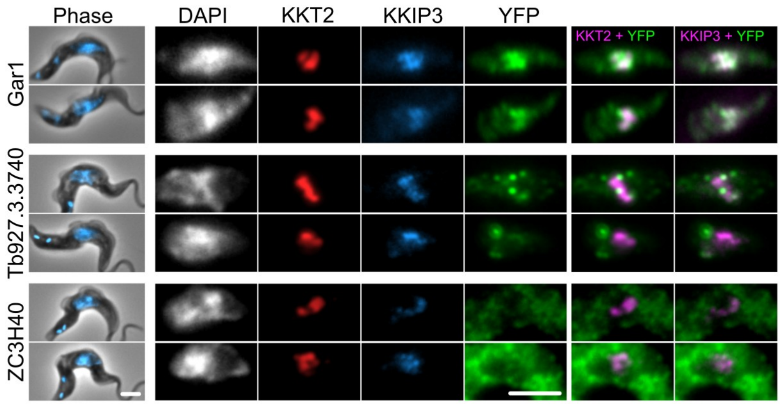

Supplementary Figure S4. KKIP-interacting proteins transiently associate with trypanosome kinetochores. Micrographs of bloodstream-form cells at metaphase expressing one of Gar1, Tb927.3.3740 and ZC3H40 endogenously tagged with YFP at the N-terminus. Markers for the inner and outer kinetochore were also tagged with mScarlet-I (KKT2) and mTurquoise2 (KKIP3), respectively. Counter-staining of DNA with DAPI is also shown. Scale bar: 2 μm.

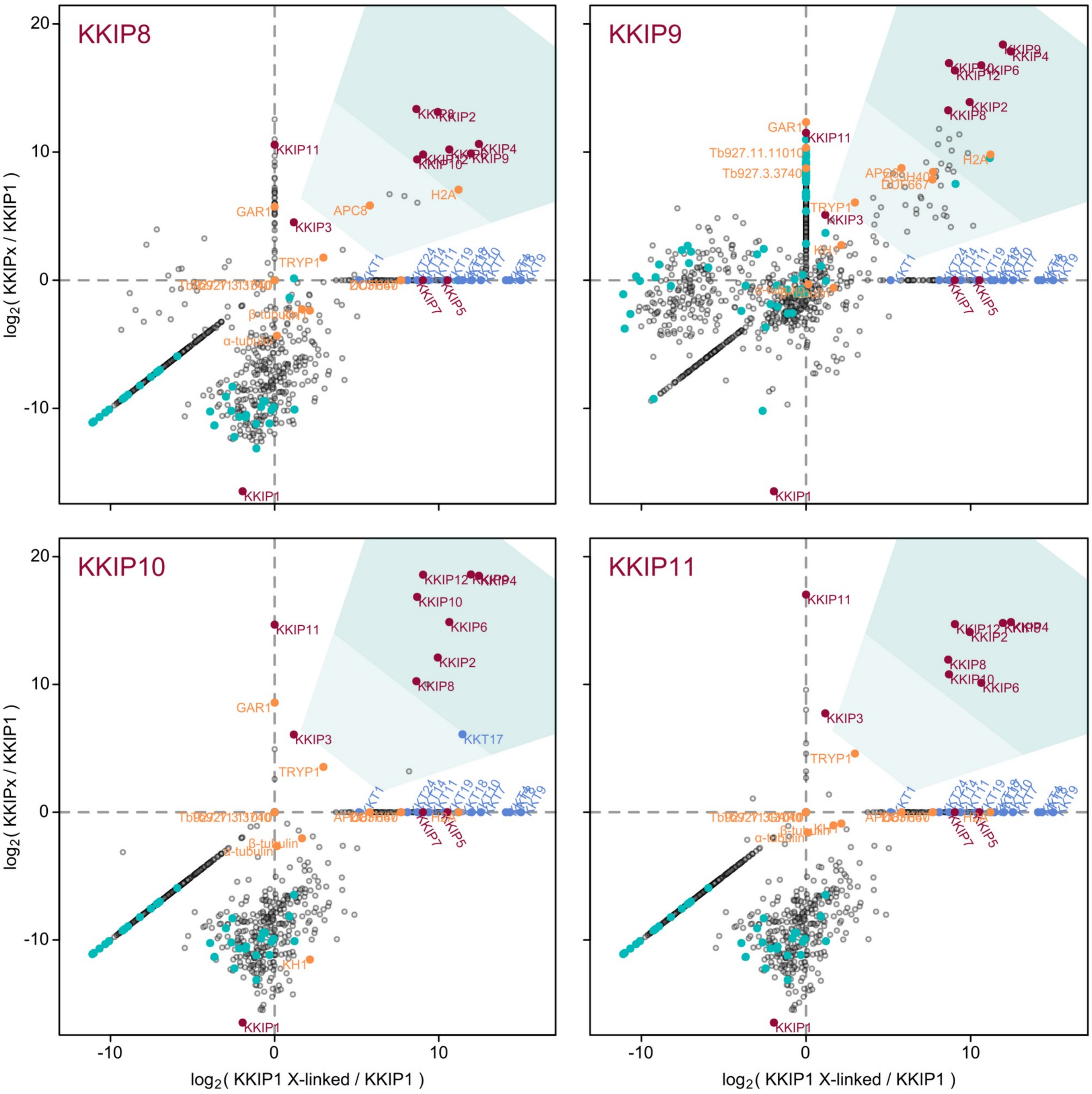

Supplementary Figure S5. KKIP8-KKIP11 interact with the stable kinetoplastid outer kinetochore. Extension of the label-free semi-quantitative mass spectrometry-based approach used in Figure 1–figure supplement 1. Proteins identified as ribosomal, KKTs and KKIPs are highlighted, in addition to proteins highly enriched in multiple experiments (neighbors).

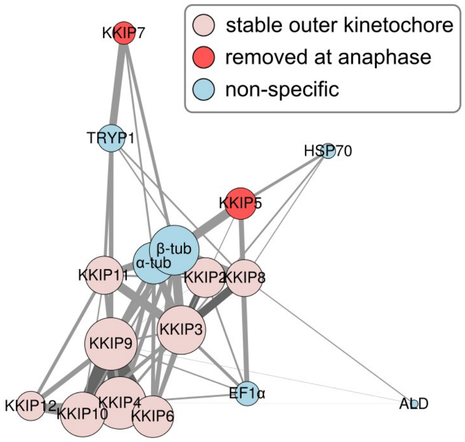

Supplementary Figure S6. Network analysis of raw protein levels from immuno-purifications. All proteins occurring in the top 25% of integrated spectral intensities (SINQ) in more than one experiment are shown. Vertex areas are scaled according to sum log SINQ value across all experiments. Edge thickness reflects sum log SINQ value for each specific interaction. Light and dark gray edges represent uni- and bi-directional hits, respectively. Vertices are colored as described in the legend.

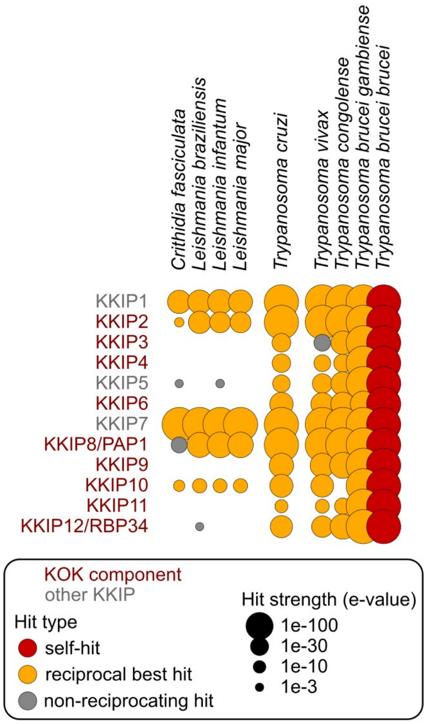

Supplementary Figure S7. Conservation of KKIPs across Trypanosomatida. Circles show strength of BLAST hits (e-values) for proteins from *Trypanosoma brucei* reference strain (*T. b. brucei* TREU927) in select organisms for which near complete genome information is publicly available (TriTrypDB). Reciprocating best hits are highlighted in orange.
